# How do psychedelics impact people with a history of non-affective psychosis? A qualitative study

**DOI:** 10.3389/fpsyt.2025.1716545

**Published:** 2025-12-09

**Authors:** Haley Maria Dourron, Heith Copes, Daniel H. Grossman, Melissa Bradley, Christopher R. Nicholas, Jessica A. Turner, Gaige Allen-Clark, Maggie Gossage, Peter S. Hendricks

**Affiliations:** 1 Center for Social and Affective Neuroscience, Department of Biomedical and Clinical Sciences, Linköping University, Linköping, Sweden; 2 Department of Psychiatry and Behavioral Neurobiology, Heersink School of Medicine, University of Alabama at Birmingham, Birmingham, AL, United States; 3 Department of Criminal Justice, University of Alabama at Birmingham, Birmingham, AL, United States; 4 Department of Psychology, University of Alabama at Birmingham, Birmingham, AL, United States; 5 Department of Family Medicine and Community Health, University of Wisconsin-Madison, School of Medicine and Public Health, Madison, WI, United States; 6 Department of Psychiatry and Behavioral Health, The Ohio State University, Columbus, OH, United States; 7 Independent Researcher, Los Angeles, CA, United States

**Keywords:** psychedelics, psychosis, schizophrenia, qualitative research, harm reduction

## Abstract

**Background:**

People with a history of psychotic disorders are excluded from contemporary trials of psychedelic-assisted therapy. Although survey studies have explored the impact of naturalistic psychedelic use on people with a history of psychotic disorders, a qualitative study has yet to examine the subjective effects of psychedelic use in this population during both the acute effects and perceived long-term impacts.

**Method:**

Two semi-structured in-depth interviews were conducted with participants (n = 19) who had used psychedelics after the diagnosis of non-affective psychotic disorders (NAPD). Interviews probed the perceived positive and negative effects of psychedelic use. Data were analyzed using reflexive thematic analysis.

**Results:**

We constructed three major themes: 1) common challenges during the acute effects of psychedelics; 2) psychosis-specific psychedelic experiences; and 3) post-acute and long-term effects. In the common challenges of psychedelic use theme, participants often described experiences with transient anxiety, which sometimes resulted in brief self-reported hospitalizations. In the psychosis-specific psychedelic experiences theme, the subthemes consisted of psychedelics during and after psychosis, self-compassion and reduced self-stigma, and insight into hallucinations and delusions. The post-acute and long-term effects theme included subthemes of positive effects, negative effects, and a lack of long-term effects. Overall, most participants described some benefits of psychedelics, but the durability of perceived benefits varied widely.

**Conclusion:**

Psychedelics might have heterogeneous impacts on people with NAPD, including both risks for harm and potential benefits. Our findings may assist in the development of safety and tolerability trials, highlighting the need for more nuanced work that examines how psychedelics impact people with NAPD.

## 1 Introduction

Psychotic disorders are complex conditions involving positive symptoms (e.g., hallucinations), negative symptoms (e.g., avolition), cognitive symptoms, and altered self-experiences (1, 2). People with a history of psychotic disorders are excluded from contemporary clinical and experimental studies using 5-HT_2A_ receptor agonist psychedelics such as psilocybin. This is due to caution around the putative risk of precipitating a psychotic episode in people predisposed to such disorders (3, 4). The rationale for this concern may lie, at least in part, in the partial overlap between the phenomenology of psychotic disorders and psychedelic-induced subjective effects (5). Despite the safety precautions present in clinical trials, psychedelics are increasingly used in uncontrolled settings (6, 7). Some older estimates report that up to 30% of people with a psychotic disorder have used a hallucinogen, a broader category of substances that includes psychedelics, in their lifetime (8, 9). Nevertheless, no prior research has qualitatively investigated the experiences of people with a history of non-affective psychotic disorder (NAPD) who have consumed a psychedelic in naturalistic, unstructured settings. Qualitative research provides a method to gain a more holistic understanding of the range of such experiences and could be instructive for a more systematic evaluation of the potential risks and benefits of psychedelic use in people with NAPD.

This study focuses on people with a history of NAPD, as classified by the DSM-5-TR (10). This subgroup of psychotic disorders was selected as the focus of our study as emerging research suggests that people with affective psychotic disorders might have distinct risks from those with NAPD (11–13). Both patient groups have been historically excluded from contemporary psychedelic trials (3), but researchers have begun to explore psychedelic-assisted therapy in people with bipolar disorder type II (14). Historically, some early studies in the 1950s and 1960s included patients with schizophrenia (15, 16). However, these early studies often failed to meet modern safety and methodological standards (15, 16). Most of these studies provided no psychological support or psychotherapy, and lacked a double-blind, placebo-controlled design, which are common components of most clinical trials today. Many early studies also used supratherapeutic doses, repeated administrations in close proximity, and relied on diagnostic categories that are no longer in use (15, 16). This makes translating the results of these early studies to modern settings challenging (17–19).

There is increasing interest in re-evaluating the strict exclusion of individuals with a history of NAPD from psychedelic-assisted therapy (PAT) trials (15, 16, 20, 21). This is likely motivated by the high comorbidity of psychotic disorders with conditions potentially responsive to PAT, including substance use disorders and post-traumatic stress disorder (22, 23). Furthermore, a multisite trial assessing the tolerability of 3,4-methylenedioxymethamphetamine.

(MDMA), an empathogen-entactogen with some psychedelic-like properties, has recently commenced in people with schizophrenia (24, 25). Nevertheless, some theories suggest psychedelics could be harmful for people with NAPD. More explicitly, relaxed beliefs under psychedelics (REBUS; 21) posits that reduced priors can occur in both psychedelic-induced states and acute psychosis. According to this theory, among people with a predisposition to NAPD, reduced priors during a psychedelic state could precipitate a psychotic episode.

Despite these theoretical concerns, empirical data on psychedelic use in people with psychotic vulnerabilities remain limited and contradictory. One longitudinal study reported a reduction in psychotic symptoms following psychedelic use (12), and a cross-sectional survey study reported an association between a greater number of psychedelic use occasions and fewer auditory hallucinations (26). Similarly, a recent survey study found that memorable psychedelic experiences were associated with improved mental health and emotional well-being in people with a history of psychotic experiences, although a minority reported more mixed outcomes (27).

More concerningly, another study reported that, in people with genetic risk for schizophrenia and bipolar disorder, psychedelic use is associated with a greater risk of manic symptoms (28). Taken together, these studies suggest psychedelics have a poorly understood impact on people with NAPD.

Nonetheless, many recent survey studies focused exclusively on psychotic symptoms, and did not delve into other aspects of participants’ well-being. Developing a more integrative understanding of how people with NAPD are impacted by psychedelic use is instrumental for designing studies that better capture the range of risks and benefits. Additionally, as more people use psychedelics, it is important to understand how those with presumably higher vulnerability to adverse effects might respond to these drugs. The present study aims to address these gaps through reflexive thematic analysis of semi-structured interviews with people who used psychedelics after having experienced a non-affective psychotic episode.

## 2 Materials and methods

Participants were recruited using criterion-based purposive sampling. Given the low base rate of people with psychotic disorders who have used psychedelics in the general population, this approach was deemed appropriate for this exploratory study, as is the norm with many phenomenological research studies (29). Digital flyers describing the study were posted on various drug and psychosis-related web forums. Those with social influence in the psychedelic community were encouraged to post flyers on their social media accounts. This sampling strategy may have led to a selection bias favoring higher-functioning, digitally literate individuals interested in psychedelics compared to those with lower-functioning or less interest in psychedelics. Nevertheless, qualitative research often aims to describe people’s experiences in depth rather than to draw generalizable conclusions (30). Flyers were posted along with a link to a screening survey hosted on Qualtrics that included an information sheet about the study. For a full list of inclusion and exclusion criteria, see **Table 1**.

**Table 1:** Study inclusion and exclusion criteria.

Exclusion criteria	Inclusion criteria
• People who have only experienced psychosis within the context of an affective disorder (e.g., bipolar disorder) • People who have experienced psychosis only within the context of another medical condition, such as epilepsy. • People who have only experienced substance-induced psychosis. • People who are currently receiving inpatient psychiatric treatment in a hospital or group home. • Volunteers must not be in an acute state of psychosis. • People who have only “microdosed” psychedelics • People who cannot commit to two interviews within 30 days of each other • People who have only used psychedelics prior to their first episode of psychosis.	• 18 years of age or over • Fluent in English • Have a history of psychosis • Could be a single episode of psychosis, or part of a non-affective illness such as schizophrenia • Have used a psychedelic at “a full dose” • Defined as a dose with strong psychoactive effects; not a subperceptual “microdose” • Have had a psychotic episode prior to the use of psychedelics • Have a device capable of video conference; have regular access to email • People who are comfortable participating in two interviews that will take place over Zoom about their most impactful experiences with both psychedelics and psychosis, and how these experiences may compare to experiences produced by other substances

### 2.1 Study procedure

A screening survey collected demographic, mental health, and drug use histories. Participants reported their country of residence, but no other geographic information was determined. To examine lifetime history of psychedelic use, participants were asked, “Have you ever used any of the following classic psychedelics at a psychedelic-level dose (not just “microdosing”),” and given a list of substances to select from during the first portion of screening. If they confirmed psychedelic use beyond microdosing, participants were then able to enter additional demographic and mental health information. Participants also self-reported whether they had a psychiatric hospitalization not involving the acute effect of a substance (yes or no). In later sections of the screening survey, to estimate lifetime psychedelic use frequency, potential participants were asked, “On how many separate occasions have you taken one or more of these classic psychedelics at a psychedelic-level dose (producing strong psychoactive effects). Please enter an exact number, even if you are unsure.” Participants were then given a checklist that included the following psychedelics: LSD (“acid”), psilocybin-containing mushrooms (“magic mushrooms”), DMT, ayahuasca, peyote or San Pedro or mescaline, 5-MeO-DMT, and analogs of the above substances (e.g., 1P-LSD, ETH-LAD, 4-AcO-DMT). Respondents with consistent response patterns who met inclusion criteria were then contacted via email to schedule an interview.

Qualified participants then participated in two semi-structured interviews conducted over Zoom. We conducted two interviews to build trust and rapport with our participants, and to reduce participant fatigue during the interview sessions (31). This also allowed us to ask more in-depth follow-up questions during the second interview and develop a richer understanding of participants’ experiences. In the first interview, participants were first read the information sheet and given the opportunity to ask questions about the study. If participants were still interested and met inclusion criteria, data collection commenced, following the outline provided in the semi-structured interview guide. The lead author conducted all interviews. The first interview explored participants’ experiences with psychosis and psychedelics separately, examining short and long-term positive and negative aspects of both experiences. Additionally, participants were asked to describe their experiences with psychotic disorders extensively, in part to build an understanding of the participant’s history and broadly confirm experiences consistent with a NAPD diagnosis. The second interview focused primarily on comparing psychedelic-induced experiences with psychosis. However, there was substantial unstructured overlap between both interviews. Participants did not provide their names, and pseudonyms are used throughout the manuscript. A copy of all survey screening questions and the semi-structured interview guide are available online at Open Science Framework (https://osf.io/asm7b). This study was approved by the Institutional Review Board at the University of Alabama at Birmingham.

### 2.2 Qualitative analysis

We used reflexive thematic analysis due to its flexibility in accommodating both experiential content and contextual factors (e.g., setting) (30, 32). Such an approach emphasizes the researchers’ roles in actively interpreting the findings using an interactive and reflexive approach that allows for a richer understanding of naturalistic psychedelic use in individuals with a history of NAPD. Reflexive thematic analysis takes a non-positivist stance that researchers’ subjectivity is a resource rather than a bias to be corrected (32). It also argues that lived experiences shape all aspects of the research process, from the questions being asked to the way data are interpreted (33).

Our interpretive stance was shaped by the researchers’ backgrounds and lived experiences. The project lead (HMD) had prior exposure to the “psychedelic community,” including volunteering to assist people having adverse effects from psychedelics in uncontrolled settings (e.g., festivals). HMD is neurodivergent, having been diagnosed with Asperger’s syndrome at an early age, and has experienced stigma related to this diagnosis. Two other authors are neurodivergent, one with a personal history of autism spectrum disorder (MB) and one with firsthand experience of a psychotic disorder (GAC). Authors varied in their firsthand experience with the effects of psychedelics, ranging from no lifetime use to more than fifty experiences. The author with lived experience of psychosis and psychedelic use (GAC) contributed to the development of the semi-structured interview guide and recruitment strategies. These perspectives informed both the study design and interpretation of findings. We recognize that other researchers might have emphasized different aspects of participants’ accounts, but we view the inclusion of researchers who are neurotypical and non-neurotypical and those with lived experience perspectives as a strength of this analysis.

Analysis used Braun and Clarke’s six-stage approach (30, 34). The lead author, who conducted the interviews, familiarized herself with the data in several steps, which began with transcription and reviewing transcripts for accuracy. She then read all the transcripts several times and reflected on initial patterns related to participants’ perceptions of psychedelics and psychosis. She then generated initial codes in NVivo14 by tagging and labeling segments that captured both semantic and latent meanings related to the broader topic (35). She and the second author then met to discuss the initial codes within the transcripts. After discussing broader patterns within the data, the lead author went back to the transcripts to continue initial coding of all the transcripts. The first and second author then met to review the initial codes and to develop broader themes that reflected shared meanings. During this stage, it became apparent that the richness of the data would result in multiple studies. Once all transcripts were coded based on these broader themes, the remaining coauthors reviewed themes and helped refine the key themes and subthemes, which are discussed here. The results were drafted by the first and second authors where they defined themes and selected illustrative quotations. The remaining authors checked and commented on the findings to ensure that data and interpretation were consistent with the research questions. Saturation and intercoder reliability were not characterized, as these are explicitly not recommended for reflexive thematic analysis (32).

### 2.3 Participant characteristics

Participants (N = 19) are described in terms of demographic, mental health history, and psychedelic drug use history characteristics in **Table 2**, **Table 3**, and **Table 4** (for non-psychedelic drug use history, see **Supplementary Table 1**). Most participants were male (78.9%), and educational attainment and employment status varied widely. This sample featured people from various countries, including the United States (n = 7, 36.8%), the United Kingdom (n = 3, 15.8%), Germany (n = 3, 15.8%), Canada (n = 2, 10.5%), Bulgaria (n = 1, 5.3%), the Czech Republic (n = 1, 5.3%), Denmark (n = 1, 5.3%), and Sweden (n = 1, 5.3%). Participants were recruited from Reddit (31.6%), Twitter (31.6%), other sources (21.1%), or a friend (15.8%). The study resulted in 31 hours and 32 minutes of interview data, averaging 50 minutes per interview, with two interviews per participant.

**Table 2:** Sociodemographic characteristics of participants.

Characteristic	n (%)
Age	
18 – 24	4 (21.1%)
25 – 34	10 (52.6%)
35 – 44	3 (15.8%)
45 – 54	1 (5.3%)
55 +	1 (5.3%)
Gender	
Male	15 (78.9%)
Female	3 (15.8%)
Other	1 (5.3%)
Race & ethnicity	
White	15 (79.0%)
Asian	4 (21%)
Hispanic	2 (10.5%)
Education	
Less than high school	1 (5.3%)
High school graduate	1 (5.3%)
Some college	6 (31.6%)
2-year degree	2 (10.5%)
4-year degree	6 (31.6%)
Professional/doctorate degree	3 (15.8%)
Employment	
Student	5 (26.3%)
Employed full or part-time	6 (31.6%)
Unemployed	3 (15.8%)
Disability	5 (26.3%)
Yearly income	
< $19,000	10 (52.6%)
$20,000 - $39,999	8 (42.1%)
> $40,000	1 (5.3%)
Sexual orientation	
Heterosexual	13 (68.4%)
Bisexual	5 (26.3%)
Other	1 (5.3%)
Religion	
Atheist/Agnostic/None	9 (47.4%)
Christian	3 (15.8%)
Buddhist	2 (10.5%)
Muslim	1 (5.3%)
Other	4 (21.1%)

Currency is in US dollars.

**Table 3:** Mental health history.

Characteristic	n (%) or M (SD)
Self-Reported Diagnosis	
Schizophrenia	6 (31.6%)
Schizoaffective Disorder	4 (21.1%)
Brief Psychotic Disorder	4 (21.1%)
Schizophreniform Disorder	1 (5.3%)
Unspecified Psychotic Disorder	4 (21.1%)
Age of Diagnosis of Psychotic Disorder	21.1 (3.7)
History of Antipsychotic Medication Use	17 (89.5%)
Non-Drug-Related Psychiatric Hospitalizations	13 (68.4%)
Family History of Psychotic Disorders	8 (42.1%)

Participants self-reported their prior diagnoses by a mental health professional, such as a licensed psychologist or psychiatrist. Participants also reported their age when first diagnosed with a psychotic disorder by a mental health professional. Self-reported psychiatric hospitalizations not involving acute substance intoxication were also reported as a yes or no.

M, mean; SD.

**Table 4:** Lifetime psychedelic drug use history.

Drug	Lifetime use n (%)	Frequency of use among users median, [Range]
Psilocybin-containing fungi	18 (94.7%)	12 [1 – 132]
LSD	17 (89.5%)	12 [1 – 50]
DMT	11 (57.9%)	3 [1 – 200]
Analogs (e.g., 1P-LSD)	8 (42.1%)	5.5 [1 – 100]
Ayahuasca	2 (10.5%)	1.0 [1]
Peyote/San Pedro	3 (15.8%)	2.5 [2 – 3]
5-MeO-DMT	3 (15.8%)	3.0 [1 – 50]

Lifetime use refers to any prior history with a substance at “psychedelic-level” doses (e.g., producing strong psychoactive effects) excluding microdosing. Among people who endorsed use of a substance, lifetime use frequency of use was calculated. Abbreviations: LSD refers to lysergic acid diethylamide; DMT refers to N,N-dimethyltryptamine; 1P-LSD refers to 1-propionyl-lysergic acid diethylamide; 5-MeO-DMT refers to 5-methoxy-N,N-dimethyltryptamine.

## 3 Results

### 3.1 Common challenges during the acute effects of psychedelics

For many participants, moments of psychedelic-induced experiences were challenging, often due to anxiety or unpleasant somatic effects. Several participants described the somatic effects of psychedelics, such as nausea and “muscle tension” (Bill) as the most salient negative acute effects of psychedelics. For some participants, anxiety was limited to the onset of drug effects, while others described more sporadic anxiety. Participants’ anxiety was often, but not always, coupled with contextual factors, such as use in an insecure environment or taking too high a dose. Still, less often some participants reported anxiety with no apparent source.

Despite reports of anxiety, participants often mentioned benefits from their experiences. Some even reminisced on transient anxiety with a sense of humor (“looking back on it was kind of funny,” Harry). Moreover, several participants insisted that they never really had a “bad trip” or saw “challenging experiences” as having benefits. For instance, Friedrich stated, “I would say I had no negative experiences, but challenging? Yes.” These reports suggest that although many had portions of their experiences that were negative, most of these effects were not perceived as severe. These sentiments parallel responses to “bad trips” that have been described in the broader population of naturalistic psychedelic users (36).

Nevertheless, four participants reported hospitalization during the acute effects of psychedelics. All four of these participants reported high doses of psychedelics and/or polysubstance use. In three of these participants, hospitalization was related to severe anxiety. These individuals reported being allowed to leave the hospital within a day or just hours after admission and denied any lasting negative aftereffects. One participant, Ivan, reported a more severe adverse response, resulting in 11 days of hospitalization, following the combined use of 4-bromo-2,5-dimethoxyphenethylamine (2C-B), N,N-dimethyltryptamine (DMT), and cannabis. Ivan had a history of traumatic brain injury, which may have contributed to a more adverse outcome (37). See the *Supplementary Materials* for detailed information regarding each reported acute hospitalization.

### 3.2 Psychosis-specific experiences

All participants described aspects of their psychedelic experiences that interplayed directly with their psychosis. Some participants expressed anxiety that using psychedelics might worsen their psychotic symptoms. Less frequently, other participants emphasized feeling unconcerned about this risk. Additionally, the content of psychedelic experiences was often shaped by participants’ history of psychosis. Most often, this included participants discussing cultivating self-compassion and reducing self-stigma surrounding their psychotic disorder diagnosis. Six people also described experiencing insight into their psychosis-related hallucinations and/or delusions during secondary to psychedelic use.

#### 3.2.1 Psychedelic experiences during and after psychosis

A slight majority of participants reported they used psychedelics both before and after recovering from an episode of psychosis. Most participants described the overall subjective effects of psychedelics as similar before and after a psychotic episode. When participants chose to use psychedelics during an acute psychotic episode, perceived effects were variable. Notably, John and Marek both reported gaining greater insight into their hallucinations consequent to psychedelic use when they were in acute psychosis. In contrast, during acute psychosis, Nick described taking high doses and feeling minimal effects, and Ian discussed taking a high dose and being hospitalized for less than 24 hours (see *Supplemental Materials*). This suggests that psychedelics might have more variable effects during acute psychotic episodes, rather than during more stable low-level symptoms or recovery.

When using psychedelics after an episode of psychosis, many participants shared they felt the need to be cautious despite noticing a lack of symptom resurgence. For example, Alex described a perceived tension between his generally positive experiences with psychedelics and an underlying feeling of potential risks:

I guess that my worry is that sometimes maybe I’m actually unknowingly crazy after all, and I’m just not smart enough to know that that’s what’s happening. Because I do see all these stories of people that seem like they’ve gone crazy on psychedelics, supposedly, or something.

Still, others denied any potential connection between their psychotic symptoms and psychedelic use. Most saliently, this was the case even for Ivan who reported 11 days of hospitalization after the use of 2C-B, DMT, and cannabis combined, but generally thought psychedelics had a positive effect on him:

I believe that the drugs have very little to do with the psychosis. I believe it’s completely natural for me. The psychedelics just helped me cope with my messed up reality in a more positive way.

These reports show that people were generally aware of the risks that psychedelic use is often believed to carry for people with psychotic disorders, but the level of concern this fostered in monitoring the potential impacts on their own mental health was variable.

#### 3.2.2 Self-compassion and reduced self-stigma

The most discussed psychosis-specific psychedelic experience among participants was increases in self-compassion and reduced internalized stigma related to their diagnosis. These experiences were often catalyzed by reflection during the acute effects of psychedelics but had a longer-term impact. As John explained:

A big thing it helped me with is not feeling ashamed about what I’ve gone through. Typically, if you tell somebody, ‘Oh, I’ve had psychosis. I’ve been crazy,’ a lot of people are going to think, ‘Oh, this person’s been crazy before. I don’t want to know this person.’ I had this idea of, ‘Am I a tainted person?’ DMT helped me come to the conclusion that I’m not. I just went through something negative. It doesn’t mean I’m ruined or worthless. I shouldn’t see myself like that. That’s the biggest thing it helped with.

Others echoed John’s report by sharing that they also felt a reduction in self-stigma, accompanied by broader increases in self-compassion and self-acceptance. For instance, Anna described the greatest benefit of using psychedelics as becoming “accepting of myself, accepting of me being a bit bonkers.” For most participants, these experiences were accompanied by positive affect.

Yet not all experiences of self-compassion came easily or felt good in the moment. For example, Merlin reported cultivating self-compassion within the context of facing how his experience with psychosis had negatively impacted his self-image:

It felt like there was a lot of trauma caused by the psychosis that magic mushrooms would bring up. And then clear out not in terms of bringing the flashbacks to my psychosis, but more like reminding me of how those things felt and how they impacted my self-image. And there’s deep wounds carved there that seriously need healing. So, magic mushrooms have been difficult. But every time that I’ve come out of the magic mushrooms state, I’ve felt less psychotic, I felt less unhinged, I felt less at risk to myself.

A similar emotional challenge was described by Ivan, who had a flashback to a suicide attempt that occurred during his first psychotic episode years prior, during the acute effects of LSD. However, despite it being difficult in the moment, he felt more compassion towards himself and his current life circumstances due to this experience.

These reflections underscore how psychedelic experiences can help individuals with NAPD confront internalized shame and stigma related to psychosis or other adversity. These experiences were often perceived as beneficial, even when they were not accompanied by positive affect during the acute effects of psychedelics. Overall, increases in self-compassion were the most meaningful effects shared by most participants.

#### 3.2.3 Insight into hallucinations and delusions

Six participants described highly meaningful experiences where they gained insight into the false nature of their hallucinations and/or delusions. For some, this awareness emerged through an internal dialogue, while for others it came as a spontaneous realization. George described gaining insight into his auditory hallucinations through an internal conversation with himself, which he clarified was not like his typical auditory hallucinations. More specifically, he said:

I just remember being told the psychosis isn’t a real thing. The symptoms are just neurotransmitters, and nothing of any relevance. It’s not real—the people I hear, or the voices—they’re not real. It was strange, because I had a conversation with myself and convinced myself the condition isn’t real.

George reported that this experience had a lasting impact on his psychotic symptoms and shared, “it wasn’t until that experience that I could distinguish between reality [and the symptoms].” John also reported a similar experience of having insight into his current mental state that prompted him to seek psychiatric care. Ultimately, this led to him being prescribed an antipsychotic medication, which in turn resulted in his recovery from psychosis, as he described:

When I did DMT during the psychosis, I had a trip that told me I need to go to the doctor, because I’m trusting my mind, and my mind being the source of information that I’m getting stuff from isn’t reliable … That’s why I went to the doctor and that’s when I got the help and I started taking antipsychotics and I’m fine now.

Sudden insights into hallucinations and delusions occurred in both positive and negative affective states during the acute effects of psychedelics. For some, these moments occurred within the context of significant anxiety. Marek became very anxious when his brother, who had been supervising him during the acute effects of LSD, left for an extended period. Nevertheless, he described the “collapse” of a delusional framework that involved interaction with several voices but left him aware that he was “talking to myself.”

Some people did not have specific insights during the acute effects but still described improved perspectives on their psychotic symptoms. Alex shared that prior to an experience, he felt like paranoid delusions about his family were “consuming every part of [his] mind.” However, after a psychedelic experience, he “didn’t really feel attached to the delusional narrative.” Similarly, Ian reported that after his experience, “I wasn’t hallucinating less afterward, so much as I was more rational in my understanding of being psychotic. It still felt real, but I knew it wasn’t real afterwards, and I needed to take stuff for it.”

For some, insight and improved perspectives remained at their full initial strength after the acute effects subsided. For Marek and John, insight into their symptoms increased gradually after the experience. In contrast, Merlin experienced insight and perspective shifts during psychedelic use that took place in the “psychosis buildup period” (e.g., early relapse), which diminished once drug effects subsided. In summary, most participants who gained insight reported that psychedelics helped them view symptoms as products of their mind, fostering a more functional relationship with them.

### 3.3 Perceived post-acute and long-term effects

Most participants described experiencing some post-acute beneficial effects. However, there was substantial variability in the duration of post-acute effects of psychedelics. Some people described positive impacts that were perceived to last for months, whereas others only noticed positive effects lasting a few days or a few weeks. Many participants described negative effects, but for most, these were short-lived and had minimal impact on their lives. Some participants reported they felt their psychedelic use had minimal lasting impact on them, in general.

#### 3.3.1 Potential positive effects

After a psychedelic experience, most participants perceived positive long-term effects, often including a sense of increased motivation, social connection, and lifestyle changes. These changes were often salient but varied in their durability. Positive changes were often interrelated. For example, Jordon said, “For about a month afterward, I wouldn’t really have any social anxiety and felt a lot less stressed out, and that made me have fewer paranoid episodes.”

For some people, a renewed sense of motivation to connect with others was an especially notable aftereffect. Marek reported, “it helped me get out of the psychosis and helped me connect with my friends more.” Carlos also described a lasting sense of feeling “more connected to other people” and improving his relationships with his family and friends. Alex remarked that his increase in social behavior seemed to be noticeable to others. As he explained, “I went back to my family [immediately after the experience], I think they definitely noticed I was acting more confident and better socially.”

Many people also described increased engagement in activities to stay well, such as more consistent medication use, reducing substance use, or engaging in hobbies more. As Sophie explained, “[the psychedelic experience] kind of makes you want to look after your needs better.” Ian shared that after an experience with escaline, a mescaline analog, he “started being more serious about getting sleep and taking my meds and stopping using drugs.” Felix also shared that psychedelic experiences inspired him to reduce his alcohol use: “I started drinking alcohol when I was 14 or 15 and drank really a lot. But through the psychedelic experiences, I saw this was damaging me long-term. I drank less and less, and then I stopped.” Still, reductions in substance use were not a universal phenomenon, and some people continued to use other substances. Notably, Ella reported returning to problematic alcohol use two days after her psilocybin experience. Nevertheless, many people did describe at least a temporary increase in motivation to engage in other wellness-maintaining activities.

#### 3.3.2 Potential negatives effects

Participants also described negative effects of psychedelic use, with most describing them as mild and short-term. Several participants were hesitant to say there were negative effects, but most, when probed, provided an answer. Nevertheless, some still reported no perceivable long-term negative effects. Oftentimes, the negative effects described were mentioned by only one person, so these are not discussed at length in this study due to our focus on describing our sample more generally.

Among the negative post-acute effects mentioned by several participants, poor sleep was frequently mentioned. Sometimes the context of use contributed to sleep disturbances. For instance, people who consumed psychedelics at night often reported not sleeping immediately after use. For Chris, this included a multiday polysubstance use occasion, including LSD, methamphetamine, opiates, and cannabis, resulting in him feeling “all tired and grumpy.” Still, sleep disturbances were not universal, and less frequently, participants remarked on motivation to have better sleep hygiene as discussed in *Potential Positive Effects*.

#### 3.3.3 Lack of long-term impact and indifference

It is worth recognizing that some participants were indifferent about their experiences and did not describe long-lasting effects, positive or negative. This was more common than reporting long-term negative effects, but not as common as noting some lasting benefits. As Ella noted, “I don’t think that it did much in one way or another.” Commonly, people who lacked long-lasting benefits still described their experiences as fun in the moment. Others reported positive effects from repeated use but had difficulty pinning changes to a specific experience. Most notably, when David was asked to describe a particularly impactful experience, he replied he could not think of one, stating, “I’ve been on so many full-on trips that they’re just like whatever.”

## 4 Discussion

The present study explored naturalistic psychedelic use in people with a history of NAPD, including psychosis-specific experiences and the perceived post-acute and long-term effects. Some challenges during the acute effects of psychedelics, including transient anxiety, were reported. However, participants reported that these outcomes were not what was most impactful. Many participants reported an increase in self-compassion as being highly meaningful. A sizeable minority of participants reported increased insight into their psychotic symptoms during the acute effects of psychedelics that persisted for some time thereafter. Participants varied in how much of a long-term impact they believed psychedelics to have, with most describing either lasting benefits or a lack of long-term impact.

One of the most salient effects of psychedelics for many participants was a reported reduction in self-stigma and an increase in self-compassion. Schizophrenia is among the most stigmatized mental health conditions (38), and many people with psychotic disorders experience high levels of internalized stigma (39–42). Internalized stigma can reduce treatment adherence and help-seeking, and can also lead to social withdrawal (43, 44). For our participants, psychedelics often facilitated a sense of self-compassion surrounding prior psychotic episodes. These effects may be similar to increases in self-compassion after psychedelic experiences reported in other studies, in both clinical trials and in naturalistic use settings (45–47).

For a minority of participants, highly salient, spontaneous insights into hallucinations and delusions occurred when using psychedelics. Some reported sudden realizations that their auditory verbal hallucinations were internally generated due to “conversations” with their hallucinations. This process shares some similarities with AVATAR therapy, which creates an externalized voice that patients interact with to gradually gain a sense of control over their hallucinations (48). These findings also align with a recent study reporting that psychedelic use occasions were associated with a reduction in auditory hallucinations in people with a history of psychotic disorders (26). Not all participants described such events. However, for those who did, these moments often had a lasting impact on their relationship to their symptoms. It is possible that participants who reported enhanced insight into their symptoms and experienced other benefits had higher metacognitive capacities than those who did not. Future studies should examine whether metacognitive abilities moderate potential benefits and risks, or whether psychedelics might improve metacognitive abilities in people with a history of NAPD (49).

Negative effects and challenging experiences commonly reported with psychedelic use in general were also discussed (36, 50). Most commonly, participants reported transient anxiety. Transient anxiety is one of the most common adverse effects in trials of PAT (51), and “panic reactions” have a long history of being documented both in research and naturalistic settings (3, 4). People with psychotic disorders are at an elevated risk of anxiety disorders (52, 53). It is possible, therefore, that people with a history of NAPD may need more support to manage anxiety during the acute effects of psychedelics, but more research would be needed. Despite transient anxiety occurring during the acute effects of psychedelics, increases in post-acute anxiety were not discussed by our sample.

Four participants reported being hospitalized during the acute effects of psychedelics. All participants who reported an acute hospitalization had a history of prior mental health-related hospitalizations. No hospitalization occurred in the absence of large doses and/or polysubstance use. These experiences emphasize the need for harm reduction efforts surrounding psychedelic use, especially cautioning against combining substances (54). It is likely that a safety and tolerability trial would not prompt as many hospitalizations due to more controlled dosing parameters, lack of polysubstance use, and better psychological support.

In terms of post-acute negative effects, the most consistently discussed issue in our sample was sleep disturbance. This parallels some of the findings in a qualitative study of the naturalistic use of psilocybin in people with bipolar disorder (11) and studies of adverse effects in clinical studies of PAT (55). This suggests this phenomenon may be a potential negative outcome associated with psychedelic use in general. Nevertheless, sleep disturbance is a notable risk for worsening of psychotic symptoms (56). Therefore, this common negative effect may be more consequential for those with a history of NAPD.

Regarding future study of psychedelics in people with a history of NAPD, a clinical trial would be safer than the patterns of naturalistic use described here. It is nonetheless possible that our participants had an unusually favorable response to psychedelics. Still, the reported increases in motivation after psychedelic use might suggest a reduction in negative symptoms. This is especially significant because negative symptoms are often more persistent and less responsive to treatment than positive symptoms and correspond to poorer quality of life (57–59). It is possible that the risk proposed by psychedelics for this population could be mitigated through more nuanced selection criteria and careful trial design. Future tolerability trials should be wary that participants with NAPD may be more likely to experience acute anxiety than other groups of patients. In both harm reduction settings and in the context of a tolerability trial, good sleep hygiene should be emphasized.

## 5 Limitations

The transferability of our study findings to other samples with NAPD may be limited due to the particulars of our purposive sample. Participants in this study reported using a variety of substances, often repeatedly, and our sample may overrepresent individuals who had especially positive experiences with psychedelics. Despite this, no part of our interviews suggested that we wanted to hear positive experiences. Our participants also reported a higher level of functioning than many people with psychotic disorders. However, as many people with a history of NAPD experience a singular psychotic episode or go into remission (60–63), our participants may have been representative of this portion of the population. It is possible that those who used psychedelics and had negative experiences had too low of a level of functioning to participate in this study. Our recruitment methods likely led to a sample that is more digitally literate, higher functioning, and with a stronger interest in psychedelics than would have been found through other sampling strategies. Additionally, diagnoses, hospitalizations, and substances used were based on self-report and, therefore, may be inaccurate.

Study participants were also disproportionately men. The gender imbalance in our sample may reflect that there are more men with NAPD, and that men are more likely to engage in the risky behavior of using a psychedelic. Consistent with this view, men are more at risk for NAPD (64), and psychedelic use is more common in men, at least within the US (7, 65). These patterns notwithstanding, the relatively low levels of women in our study may have influenced the results, particularly in relationship to self-compassion and self-stigma, which can vary by gender (66, 67).

It is also possible that positive expectancies could have contributed to participants’ perceived beneficial effects. A notable instance of this previously occurring with psychedelics is microdosing (68), which may largely impact well-being due to positive expectancy effects (69–71). Despite the potential for positive expectancies, most participants acknowledged the presumed negative impact psychedelics could have on psychotic symptoms. This suggests that, in some respects, participants likely went into their experiences with negative expectancies. Still, unduly positive expectancies remain an ongoing concern for all studies of psychedelics in humans (71).

## 6 Conclusion

Our results reveal that the relationship between psychedelic use and NAPD is significantly more complex than prior work has stipulated. In our study, many people with a history of NAPD reported perceived benefits from the use of psychedelics. However, there are also clear risks of potential harms, including hospitalizations. Future work should explore specific phenomena described here, such as insight into hallucinations during experiences, in greater depth. A safety and feasibility study is needed to adequately reevaluate the existing safety exclusion criteria, particularly given the very heterogeneous nature of responses among our participants. For now, the clinical use of psychedelics in people with NAPD remains contraindicated pending positive results in a controlled safety and tolerability trial.

## Data Availability

Due to the sensitive nature of topics discussed, participants’ full transcripts are withheld to protect participants’ privacy. Requests to access the datasets should be directed to haley.dourron@liu.se.
