# Supplementary material for: How do psychedelics impact people with a history of non-affective psychosis? A qualitative study
Source: Front Psychiatry. 2025 Dec 9;16:1716545. doi: 10.3389/fpsyt.2025.1716545 (PMC12722819; doi:10.3389/fpsyt.2025.1716545)
Supplement: Supplementary file 1 [file DataSheet1.docx]

Supplementary Material

**Acute Psychedelic-Related Hospitalizations**

***Ivan.*** Ivan reported the most severe hospitalization during the acute effects of psychedelics. For background, Ivan described being diagnosed with schizophrenia as an adolescent, after a suicide attempt. Ivan also discussed a history of domestic violence experienced as a child. He reported experiencing a traumatic brain injury (TBI) as a child. He had a history of four non-psychedelic-related mental health hospitalizations totaling between 4 to 6 months.

Ivan described combining cannabis, 2C-B, and DMT with a friend. He did not provide an estimated dosage. According to Ivan, the experience produced a sense of a break in reality:

Everything was going good. I started to hallucinate a little bit and then I just had a complete shift and break in reality. I started to see angels and the statues were talking to me and I was in the park, and everything was just different and absorbing. It was a very powerful experience, and I still don't know what exactly [it] was…It was a complete break in my in my reality, everything changed.

The exact circumstances are unclear, but according to Ivan, his friend apparently called the police on him, and they took him to a psychiatric hospital. He described experiencing severe cognitive symptoms after this experience:

My symptoms after that incident were very bad. I started to forget basic words. I couldn't form sentences I was very confused and delirious all the time. It was a pretty bad moment for me. I was looking at sentences and the sentences were running away from me in a dyslexia style. Everything just crashed and I felt mentally crippled.

He was released from the hospital in under two weeks after admission, after being given “anti-anxiety drugs.” Ivan described doing psychedelics again after this incident without perceived negative effects. His experiences may have been influenced by his history of TBI and likely PTSD symptoms (e.g., from adverse childhood experiences). His experience with language difficulties, especially the aphasia-like experiences, is more typical of TBI-related deficits than psychotic disorders not related to a TBI (1–3). Another instance of a psychotic episode co-occurring with repeated polysubstance use, including psychedelics, in a person with a TBI has also been reported (4). His experience during the hospitalization was highly atypical within the context of the rest of our sample. Other experiences that Ivan shared are more in concordance with the rest of our sample and are quoted in the main text.

***Ian.*** Ian reported a diagnosis of schizoaffective disorder and fibromyalgia. He described having experienced “several” prior hospitalizations, each lasting two to three months. During one psychotic episode, he reportedly ingested “8 tabs of AL-LAD,” a lysergamide similar to LSD, which led to intense anxiety (Blumenberg & Hendrickson, 2020). As a result, he sought help from his mother, whom he lived with. As Ian shared:

The one time I got temporarily admitted to [the] hospital on a psychedelic [was] because I ended up asking my mom for help. And then she drove me to the hospital, and I was temporarily certified. But I was agitated and want[ed] to leave. The weird thing was, is that he [a medical professional] gave me a dose of IM [intramuscular] ketamine for a drug-agitated state…I pretty much just passed out after a short while after my vision kind of went weird [from being administered ketamine]…And then, when I woke up, I was doing so much better that I was able to be discharged.

Ian did not report any long-term negative effects from this experience and described more recent positive experiences with psychedelics. This hospitalization highlights how high doses of psychedelics may increase the risk of agitation. The reportedly positive impact of ketamine, a drug known to worsen psychotic symptoms based on prior tolerability studies, warrants further consideration (6). Negative effects of ketamine may **only be found** in subanesthetic doses, though. Ian likely received an anesthetic dose.

***David.*** David was diagnosed with schizophrenia and described many years of intermittent hospitalizations for mental health reasons. His longest hospital stay was approximately six months. He also reported previously living in group housing for psychiatric conditions for several years.

David reported taking 5 grams of psilocybin-containing mushrooms—higher than his typical dose—and experiencing substantial anxiety. This anxiety appeared to be due to a precautionary concern about the potential adverse effects related to his mental health history. More specifically, he shared:

I didn’t have a particularly bad trip, but I just felt very uneasy. I got a taxi and went to the mental hospital and asked if I could sleep there for the night, just in case. They said, ‘Yeah, that’s fine.’ The next day I woke up and felt awesome. I felt really great. And yeah, that was my bad trip.

He also clarified that during this time, the main reason he attended the hospital was in part due to a lack of social support elsewhere:

I was alone… there were no other people I could contact or be with during that time. So, the only option I had, if I was going to be in a place that was safe, it would be the mental hospital. That's why.

David’s experience highlights the risks of taking high doses and emphasizes the importance of having resources available for psychological support. Nonetheless, he reported no long-term effects from this experience.

***Bill.*** Bill was diagnosed with schizophreniform disorder and reported one episode of psychosis. He reported one prior mental health hospitalization of under two weeks, followed by a good response to antipsychotic medication. He described going to the hospital during the effects of psychedelics due to concerns about "a physical issue" that the consumption of a psychedelic had exacerbated his anxiety about. He had consumed “three hits of acid and 2C-P together” (6 to 8 mg). According to Bill, no physical cause was found, and he was able to leave the hospital a few hours later while still experiencing drug effects. Bill said, “Most of it was just sitting in the waiting area of the ER. But yeah, I remember we got done, and it was it was like evening, it was a beautiful sunset, and I was still tripping.” Furthermore, when asked if he believed this experience related to psychosis, he shared:

No, it wasn't a mental issue at all. I was really calm the whole time. I was actually really enjoying the trip part of the trip, like I really like being on acid. But I just had this intense pain that I felt like was an emergency.

He reported some “embarrassment” about the experience, but no notable long-term negative effects. It is unclear how much Bill’s negative somatic experience was related to his prior psychotic episode.

**Supplemental References**

1. Fujii D, Ahmed I, Hishinuma E. A Neuropsychological Comparison of Psychotic Disorder Following Traumatic Brain Injury, Traumatic Brain Injury Without Psychotic Disorder, and Schizophrenia. J Neuropsychiatry Clin Neurosci. 2004 Aug;16(3):306–14.

2. McAllister TW. Neurobiological consequences of traumatic brain injury. Dialogues Clin Neurosci. 2011 Sep;13(3):287–300.

3. Vas AK, Chapman SB, Cook LG. Language impairments in traumatic brain injury: a window into complex cognitive performance. Handb Clin Neurol. 2015;128:497–510.

4. Sami MB, Rabiner EA, Bhattacharyya S. Does cannabis affect dopaminergic signaling in the human brain? A systematic review of evidence to date. Eur Neuropsychopharmacol. 2015 Aug 1;25(8):1201–24.

5. Blumenberg A, and Hendrickson RG. A letter reporting a case of fatal ventricular dysrhythmia associated with the LSD analog AL-LAD. Clin Toxicol. 2020 Feb 1;58(2):143–5.

6. Lahti A. Subanesthetic Doses of Ketamine Stimulate Psychosis in Schizophrenia. Neuropsychopharmacology. 1995 Aug;13(1):9–19.
