# Supplementary material for: How do psychedelics impact people with a history of non-affective psychosis? A qualitative study
Source: Front Psychiatry. 2025 Dec 9;16:1716545. doi: 10.3389/fpsyt.2025.1716545 (PMC12722819; doi:10.3389/fpsyt.2025.1716545)
Supplement: Supplementary file 2 [file Table1.docx]

**Supplemental Table 1**

*Non-Psychedelic Substance Use History*

| **Substance** | ***Lifetime***  ***n* (%)** | ***Year***  ***n* (%)** | **Month**  ***n* (%)** |
| --- | --- | --- | --- |
| Cannabis | 19 (100.0%) | 11 (57.9%) | 6 (31.6%) |
| Alcohol | 19 (100.0%) | 17 (89.5%) | 8 (42.1%) |
| Tobacco/nicotine | 17 (89.5%) | 12 (63.2%) | 11 (57.9%) |
| Dissociative anesthetics | 15 (78.9%) | 6 (31.6%) | 0 (0.0%) |
| MDMA | 14 (73.7%) | 6 (31.6%) | 1 (5.3%) |
| Sedatives | 14 (73.7%) | 5 (26.3%) | 2 (10.5%) |
| Prescription stimulants | 11 (57.9%) | 4 (21.1%) | 3 (15.8%) |
| Opiates & Opioids | 10 (52.6%) | 3 (15.8%) | 1 (5.3%) |
| Major stimulants | 7 (36.8%) | 4 (21.1%) | 2 (10.5%) |
| Deliriants | 6 (31.6%) | 0 (0.0%) | 0 (0.0%) |
| Salvia | 5 (26.3%) | 0 (0.0%) | 0 (0.0%) |
| Bath Salts (Cathinones) | 3 (15.8%) | 1 (5.3%) | 0 (0.0%) |
| Synthetic Marijuana | 3 (15.8%) | 1 (5.3%) | 0 (0.0%) |
| *Amanita muscaria* | 2 (10.5%) | 1 (5.3%) | 0 (0.0%) |

***Note.*** Dissociative anesthetics include ketamine and dextromethorphan. MDMA refers to 3,4-methylenedioxymethamphetamine. Sedatives include benzodiazepines and barbiturates. Deliriants include diphenhydramine (used recreationally) and plant-based substances (e.g., belladonna). Prescription stimulants were not used as prescribed.
